# Direct Second Autologous Stem Cell Transplantation Versus Re-Induction Followed by Transplantation in Relapsed Multiple Myeloma: A Multicenter Real-World Study

**DOI:** 10.3390/jcm15135045

**Published:** 2026-06-28

**Authors:** Taha Ulutan Kars, Hakan Göker, Haluk Demiroğlu, Ümit Yavuz Malkan, Elifcan Aladağ Karakulak, Fatma Tuğba Erdekli, Burak Deveci, Süreyya Yiğit Kaya, Leylagül Kaynar, Asena Dikyar, Sait Emir Şahin, Metin Bağcı, Buğra Sağlam, Ahmet Kürşad Güneş, Ömür Gökmen Sevindik, Volkan Karakuş

**Affiliations:** 1 Division of Hematology, Konya City Hospital, 42040 Konya, Türkiye; 2 Division of Hematology, Faculty of Medicine, Hacettepe University, 06100 Ankara, Türkiye; 3 Division of Hematology, Medstar Hospital, 06100 Antalya, Türkiye; 4 Division of Hematology, Faculty of Medicine, Medipol University, 34083 İstanbul, Türkiye; 5 Division of Hematology, Etlik City Hospital, 06100 Ankara, Türkiye; 6 Department of Internal Medicine, Antalya Training and Research Hospital, 06100 Antalya, Türkiye; 7 Division of Hematology, Medical Point Hospital, 27584 Gaziantep, Türkiye; 8 Division of Hematology, Florence Nightingale Hospital, 34083 İstanbul, Türkiye; 9 Division of Hematology, Antalya Training and Research Hospital, 06100 Antalya, Türkiye

**Keywords:** multiple myeloma, autologous transplantation, re-induction, relapse, salvage therapy, survival

## Abstract

**Background**: Whether patients with relapsed multiple myeloma (MM) should proceed directly to a second autologous stem cell transplantation (ASCT2) or receive re-induction chemotherapy beforehand remains uncertain. In real-world practice, treatment allocation is highly heterogeneous, and comparative data addressing the impact of pre-ASCT2 strategy on survival outcomes are limited, partly because ASCT2 is reserved for selected patients and is not routinely performed in all relapsed MM cases. We aimed to evaluate real-world outcomes of ASCT2 performed either directly at relapse or following re-induction chemotherapy in patients with relapsed MM, with a focus on progression-free survival after ASCT2 (PFS2) and overall survival (OS). **Methods**: We conducted a multicenter retrospective cohort study including 42 patients with relapsed MM who underwent ASCT2 between 2012 and 2024 across eight centers. Patients were grouped according to pre-ASCT2 management: those proceeding directly to ASCT2 without additional systemic therapy (direct-ASCT2, *n* = 21) and those receiving ≥ 1 line of salvage chemotherapy prior to ASCT2 (re-induction, *n* = 21). Survival outcomes were estimated using Kaplan–Meier methods and explored using multivariable Cox regression analyses. **Results**: The median PFS2 for the entire cohort was 19.7 months (95% CI, 7.8–31.6), with no statistically significant difference between the direct-ASCT2 and re-induction groups (21.3 vs. 13.8 months; *p* = 0.790). Median OS was 48.2 months (95% CI, 41.3–55.1). Although OS was significantly longer in the unadjusted analysis (51.7 vs. 39.6 months; *p* = 0.019), this difference was not maintained after multivariable adjustment. Post-ASCT2 response rates were comparable between groups, and day-100 transplant-related mortality was low at 2.4%. **Conclusions**: In this multicenter real-world cohort, pre-ASCT2 re-induction chemotherapy was not associated with a clear improvement in PFS2 compared with proceeding directly to ASCT2. Observed differences in unadjusted OS likely reflect confounding by indication and selection bias inherent to retrospective treatment allocation. These findings should therefore be interpreted as hypothesis-generating, but they add to the limited real-world evidence base on ASCT2, a salvage strategy that is typically applied to highly selected patients.

## 1. Introduction

Relapsed multiple myeloma (MM) remains a therapeutic challenge despite advances in proteasome inhibitors, immunomodulatory drugs, and monoclonal antibodies. For eligible patients, a second autologous stem cell transplantation (ASCT2) remains an established salvage option, particularly for those who previously derived a clinically meaningful benefit from first autologous transplantation (ASCT1) [1,2]. Several retrospective studies, registry analyses, and contemporary real-world cohorts have demonstrated that ASCT2 can provide meaningful disease control, with median progression-free survival generally ranging from approximately 9 to 20 months and overall survival frequently exceeding three years in appropriately selected patients [3,4,5,6,7,8,9]. However, ASCT2 is not a universally applied salvage strategy in relapsed MM. In routine practice, it is generally reserved for carefully selected patients with adequate performance status, preserved organ function, available stem cell reserve, and a clinically meaningful response duration after ASCT1. As a result, even real-world studies focusing specifically on ASCT2 often include relatively limited patient numbers, particularly when analyses are restricted to non-tandem ASCT2 performed at relapse.

However, the optimal clinical approach before ASCT2 is not well defined. A key uncertainty in current practice is whether patients should proceed directly to ASCT2 at relapse or receive re-induction chemotherapy to achieve cytoreduction prior to transplantation. While re-induction is frequently used to deepen response and potentially improve post-transplant outcomes, its benefit remains unproven. Evidence directly addressing the value of re-induction before salvage autologous transplantation remains limited. In a large retrospective single-center analysis, Miller et al. compared patients who received re-induction before salvage ASCT with those who proceeded directly to transplantation and found no significant difference in post-transplant time to next therapy or OS. However, that cohort was not restricted exclusively to patients undergoing a true second transplantation after a previous ASCT, and treatment allocation remained subject to physician selection and confounding by indication [10]. Consequently, evidence specifically comparing direct ASCT2 with re-induction followed by ASCT2 remains sparse.

Furthermore, prognostic factors that inform initial therapy, such as ISS stage, early relapse after ASCT1, and depth of response, may have diminished predictive value in the salvage transplant setting. Prior ASCT2 series have identified several potential prognostic factors, including the duration of remission after ASCT1, age, number of prior treatment lines, disease status before transplantation, and depth of response after ASCT2 [3,6,8,9]. However, the prognostic contribution of these variables has not been consistent across studies, particularly in the context of different pre-ASCT2 treatment strategies. Yet few studies have evaluated these parameters comprehensively in the context of pre-ASCT2 treatment strategy.

In this multicenter retrospective study, we aimed to directly compare outcomes of patients undergoing ASCT2 either directly at relapse or after receiving re-induction chemotherapy. Our primary objective was to assess the impact of pre-ASCT2 treatment strategy on progression-free survival after ASCT2 (PFS2) and OS. Secondary objectives included evaluating post-ASCT2 response rates and transplant-related mortality and exploring the associations of ISS stage and early relapse after ASCT1 with PFS2 and OS. This study addresses a key evidence gap and provides real-world data to guide decision-making regarding the optimal sequencing of salvage therapy before ASCT2.

## 2. Materials and Methods

### 2.1. Study Design and Population

We conducted a multicenter retrospective cohort study across eight hematology centers in Türkiye, including adult patients with relapsed MM who underwent ASCT2 between 2012 and 2024. Patients were eligible if they (1) had received a prior ASCT1, (2) experienced disease relapse based on IMWG criteria, and (3) underwent ASCT2 with melphalan conditioning. Patients were excluded if they lacked complete survival data or received tandem ASCT as part of initial therapy.

The study cohort was assembled from patients who ultimately underwent ASCT2 and was not designed as a relapse-based inception cohort of all patients considered for salvage transplantation. Patients who received salvage systemic therapy but did not subsequently proceed to ASCT2 were outside the study sampling frame and were not systematically captured. Accordingly, their number and clinical outcomes could not be reliably determined from the available transplant-recipient database.

### 2.2. Pre-ASCT2 Treatment Strategy Definitions

Patients were categorized into two groups based on management at relapse:

- Direct ASCT2 group: Patients who proceeded directly to ASCT2 following biochemical or clinical relapse, without receiving additional systemic therapy after relapse.
- Re-induction group: Patients who received ≥1 line of salvage chemotherapy prior to ASCT2 with the intent of achieving cytoreduction or improving disease burden before transplantation. Salvage regimens included proteasome inhibitor, IMiD, or anti-CD38-based combinations, selected at physician discretion.

Response to re-induction therapy was evaluated prior to stem cell mobilization or conditioning. Disease status immediately before ASCT2 was defined according to the last available IMWG assessment before conditioning. For patients in the re-induction group, this corresponded to the response documented after re-induction therapy. All patients in the direct-ASCT2 group proceeded to transplantation with progressive disease, as no intervening systemic therapy was administered between documented relapse and ASCT2.

The choice between proceeding directly to ASCT2 and administering re-induction therapy before ASCT2 was not governed by a predefined protocol. Treatment decisions were individualized by the treating physicians according to the clinical pattern and tempo of relapse, disease burden, the perceived need for cytoreduction, duration of response after ASCT1, prior treatment exposure and response, patient fitness, organ function, stem-cell availability, and institutional practice. However, the specific patient-level rationale underlying treatment allocation was not uniformly documented in the medical records and therefore could not be systematically incorporated into the comparative or multivariable analyses.

### 2.3. Data Collection

Baseline clinical characteristics, ISS stage, prior treatment history, response to ASCT1, time to relapse after ASCT1, conditioning regimen, peri-transplant factors, and post-ASCT2 outcomes were extracted from institutional records. Conventional cytogenetic and fluorescence in situ hybridization (FISH) results were collected separately when available. Owing to the retrospective multicenter design and the prolonged study period, these assessments were not uniformly available across the participating centers, and complete information regarding the FISH panels, reporting thresholds, and clonal fractions was not consistently documented. Therefore, available individual FISH abnormalities were reported descriptively, but a uniform cytogenetic risk classification could not be assigned to the entire cohort. For descriptive analyses, classical high-risk FISH features were defined as del(17p), t(4;14), or t(14;16). Gain or amplification of 1q was reported separately because complete information regarding copy number, reporting thresholds, and clonal fraction was not uniformly available across centers. The distribution of FISH availability, any detected abnormality, classical high-risk features, and observed individual abnormalities was reported for the overall cohort and according to pre-ASCT2 treatment strategy.

### 2.4. Response and Outcome Assessment

Responses were assessed according to the International Myeloma Working Group (IMWG) criteria. Post-ASCT2 response evaluation was performed on day +100 and subsequently at routine clinical follow-up visits.

After the day +100 assessment, patients were followed according to routine clinical practice at the participating centers. Because of the retrospective multicenter design, follow-up intervals were not mandated by a uniform study protocol. Disease status was assessed using clinical evaluation and available laboratory and imaging findings in accordance with IMWG criteria. Dates of progression, death, and last clinical contact were obtained from institutional medical records. Patients without documented progression or death were censored at the date of their last available follow-up.

PFS2 was defined as the time from the date of stem cell reinfusion at ASCT2 to documented disease progression or death from any cause, whichever occurred first. OS was defined as the time from ASCT2 to death from any cause. For both treatment groups, the date of stem cell reinfusion at ASCT2 was used as the common time origin for OS. Transplant-related mortality (TRM) was defined as death within 100 days after ASCT2 that was not attributable to disease progression.

### 2.5. Statistical Analysis

Continuous variables were analyzed using Mann–Whitney U or Student’s t-test as appropriate; categorical variables using χ^2^ or Fisher’s exact test. Kaplan–Meier curves were compared using the log-rank test.

Variables included in multivariable Cox proportional hazards models were selected based on the following: (1) clinical relevance (ISS stage, early relapse after ASCT1), (2) *p* < 0.10 in univariate analysis, or (3) known prognostic significance in prior literature.

Based on clinical relevance and prior literature, the multivariable Cox models included pre-ASCT2 treatment strategy, ISS stage, and early relapse after ASCT1 (<18 months). Hazard ratios (HRs) and 95% confidence intervals (CIs) were reported. A two-sided *p* < 0.05 was considered statistically significant. Statistical analyses were performed using SPSS version 25.0. As a sensitivity analysis, PFS2 and OS were re-evaluated in the subset of patients with available FISH data. Survival distributions were compared using the Kaplan–Meier method and log-rank test, and univariable Cox proportional hazards models were used to estimate the association between pre-ASCT2 treatment strategy and each survival outcome. Because of the small size of this subset, the limited number of events, and the sparse distribution of high-risk abnormalities, no multivariable model incorporating cytogenetic risk was fitted.

Given the limited sample size and number of progression and death events, the multivariable analyses were considered exploratory and were intended to identify potential associations rather than establish definitive causal relationships. Accordingly, the models were intentionally kept parsimonious to reduce the risk of overfitting. Post-ASCT2 maintenance therapy was not included as a fixed baseline covariate in the primary Cox models because it represented a post-transplant exposure initiated after the start of the PFS2 and OS observation periods; treating it as a baseline variable could therefore introduce immortal-time bias. Among patients who received maintenance after ASCT2, the distribution of maintenance classes between treatment groups was compared using the Fisher–Freeman–Halton exact test. Regimen-specific survival models or analyses restricted to individual maintenance classes were not performed because maintenance was a post-baseline exposure, treatment initiation dates and subsequent modifications were not uniformly available, and the resulting subgroups and event counts were too small to support reliable time-dependent or regimen-specific analyses.

Because this retrospective cohort included all eligible patients who underwent ASCT2 during the predefined study period, no a priori sample-size calculation was performed. To quantify the statistical limitations imposed by the available number of events, an event-based post hoc minimum detectable effect analysis was conducted using the Schoenfeld approximation. Assuming a two-sided alpha level of 0.05, 80% power, and equal allocation between the treatment groups, the 26 observed PFS2 events provided sufficient power to detect only hazard ratios of approximately 3.00 or greater, or 0.33 or lower in the opposite direction. Similarly, the 11 observed deaths provided sufficient power to detect only hazard ratios of approximately 5.42 or greater, or 0.18 or lower. This analysis was used to characterize the magnitude of detectable effects and not to infer equivalence from nonsignificant comparisons.

No imputation was performed for missing data. Descriptive and between-group analyses were conducted using available-case data for each variable. Complete survival data were required for study inclusion; therefore, all 42 patients were included in the primary PFS2 and OS analyses. Complete-case analysis was used for the multivariable Cox models, and the variables included in these models were available for all analyzed patients. Variables with substantial missingness, particularly conventional cytogenetic and FISH data, were reported descriptively but were not included in the multivariable models. The number of evaluable patients or the “not available” category was reported where applicable.

### 2.6. Ethical Approval

All procedures were performed in accordance with the principles of the Declaration of Helsinki. The study was approved by the Scientific Research Ethics Committee of Antalya Training and Research Hospital (approval code: 14/11; approval date: 28 August 2025), and informed consent was waived due to the retrospective nature of the analysis.

## 3. Results

### 3.1. Patient Characteristics

A total of 42 patients who underwent ASCT2 were included. Baseline and disease characteristics according to pre-ASCT2 treatment strategy are summarized in Table 1. Early relapse after ASCT1, defined as PFS1 < 18 months, was more frequent in the direct-ASCT2 group than in the re-induction group (15/21 [71.4%] vs. 7/21 [33.3%], respectively; Fisher’s exact *p* = 0.029). The distribution of best response before ASCT1 did not differ significantly between the direct-ASCT2 and re-induction groups (Fisher–Freeman–Halton exact *p* = 0.934).

Conventional cytogenetic results were available for 17 of 42 patients (40.5%), whereas FISH results were available for 18 patients (42.9%). FISH data were available for 7 of 21 patients (33.3%) in the direct-ASCT2 group and 11 of 21 patients (52.4%) in the re-induction group.

Among the 18 patients with evaluable FISH data, an abnormality was identified in 4 patients (22.2%): 2 of 7 patients (28.6%) in the direct-ASCT2 group and 2 of 11 patients (18.2%) in the re-induction group. The observed abnormalities were not mutually exclusive. In the direct-ASCT2 group, both patients with abnormal FISH findings had del(13q) and del(17p), and one of these patients also had t(14;16). In the re-induction group, one patient had del(13q), whereas the other had gain or amplification of 1q. Accordingly, classical high-risk FISH features, defined as del(17p), t(4;14), or t(14;16), were present in 2 of 7 evaluable patients (28.6%) in the direct-ASCT2 group and in none of the 11 evaluable patients in the re-induction group. Because FISH results were unavailable for more than half of the cohort and testing panels, reporting thresholds, and clonal fractions were not uniformly documented, a reliable cytogenetic risk classification could not be assigned to the entire cohort.

Immediately before ASCT2, all 21 patients in the direct-ASCT2 group had progressive disease because they proceeded to transplantation without intervening systemic therapy. In the re-induction group, the pre-ASCT2 disease status was CR in 1 patient (4.8%), VGPR in 9 (42.9%), PR in 4 (19.0%), and PD in 7 (33.3%). The corresponding distributions are presented in Table 2.

### 3.2. Post-ASCT2 Response and Early Outcomes

The overall response rate after ASCT2 was 92.9%, with 61.9% of patients achieving VGPR or better, including 11.9% who achieved CR; no significant differences were observed between the treatment groups. Day-100 transplant-related mortality was 2.4% (1/42).

Maintenance therapy after ASCT2 was administered to 25 of 42 patients (59.5%), including 13 of 21 patients (61.9%) in the direct-ASCT2 group and 12 of 21 patients (57.1%) in the re-induction group, with no significant difference between the groups (Fisher’s exact *p* = 1.000). Among maintenance recipients, proteasome inhibitors, immunomodulatory drugs, and anti-CD38 antibodies were used in 9 (36.0%), 12 (48.0%), and 4 (16.0%) patients, respectively. Among maintenance recipients, the overall distribution of maintenance classes did not differ significantly between the direct-ASCT2 and re-induction groups (Fisher–Freeman–Halton exact *p* = 0.661). The group-specific distribution of maintenance regimens is presented in Table 2.

### 3.3. Progression-Free Survival After ASCT2

**Figure 1:**
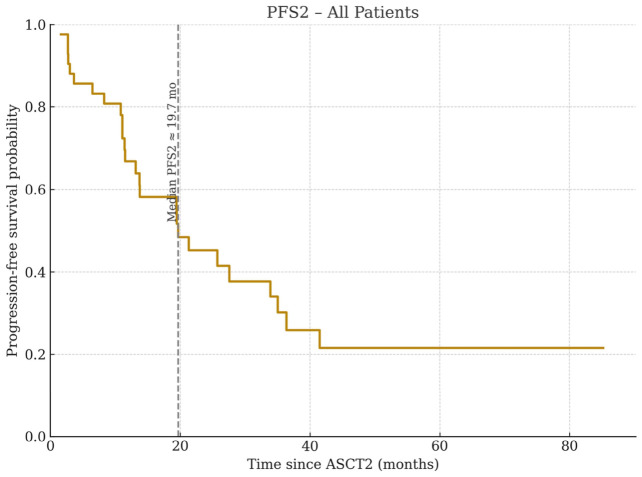
Kaplan–Meier estimates of progression-free survival after ASCT2.

**Figure 2:**
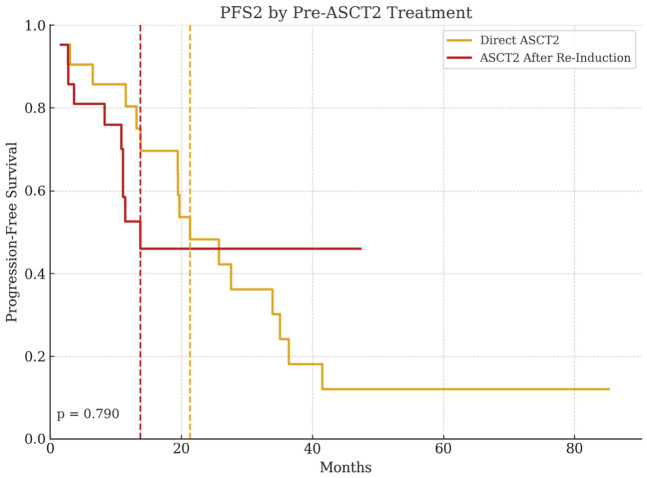
Progression-Free Survival by pre-ASCT2 Treatment Status. The colored vertical dashed lines indicate the median PFS2 for the corresponding treatment groups, with colors matching the Kaplan–Meier curves.

For the entire cohort, the median PFS2 was 19.7 months (95% CI, 7.8–31.6) (Figure 1). PFS2 was similar between the direct-ASCT2 and re-induction groups (21.3 vs. 13.8 months, log-rank *p* = 0.790) (Figure 2).

In the multivariable Cox model adjusting for ISS stage and early relapse after ASCT1 (<18 months), pre-ASCT2 treatment strategy did not independently predict PFS2 (HR 1.17, 95% CI 0.45–3.07; *p* = 0.751). Neither ISS stage nor early relapse retained statistical significance (Table 3).

### 3.4. Overall Survival After ASCT2

**Figure 3:**
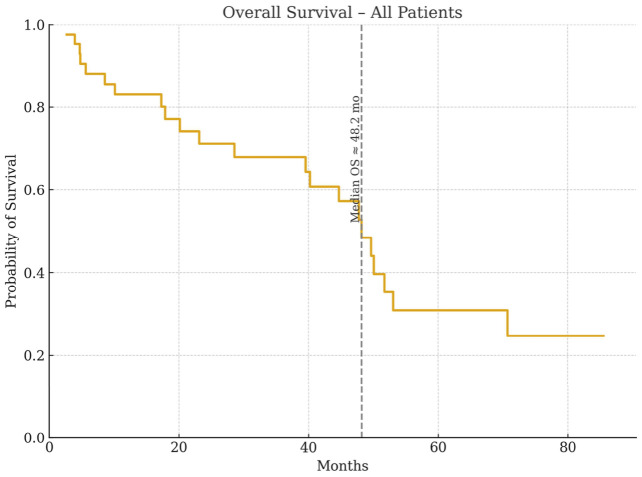
Kaplan–Meier estimates of overall survival after ASCT2.

**Figure 4:**
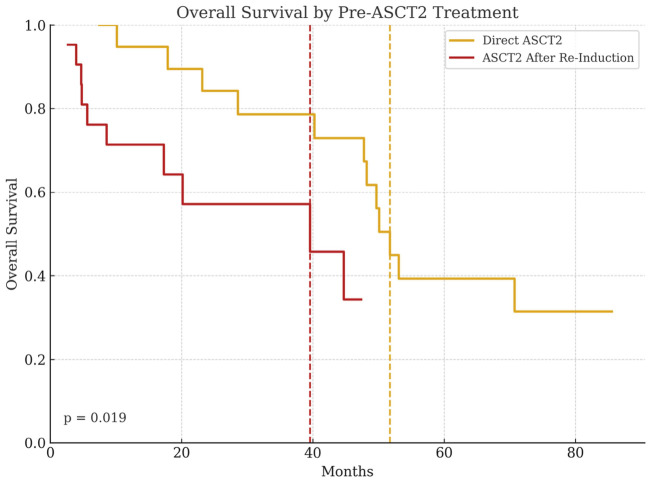
Overall Survival after ASCT2 by pre-ASCT2 Treatment Status. The colored vertical dashed lines indicate the median overall survival for the corresponding treatment groups, with colors matching the Kaplan–Meier curves.

At a median follow-up of 45 months, the median OS for the cohort was 48.2 months (95% CI, 41.3–55.1) (Figure 3). Patients in the direct-ASCT2 group had significantly longer OS than those receiving re-induction (51.7 vs. 39.6 months; log-rank *p* = 0.019) (Figure 4). However, this difference did not remain statistically significant in the multivariable analysis. After adjustment for ISS stage and early relapse after ASCT1, direct ASCT2 versus re-induction was not independently associated with OS (HR 0.41, 95% CI, 0.11–1.45; *p* = 0.164). Neither ISS stage nor early relapse independently predicted OS (Table 4).

### 3.5. Sensitivity Analysis in Patients with Available FISH Data

The sensitivity analysis included 18 patients with available FISH results: 7 in the direct-ASCT2 group and 11 in the re-induction group. Thirteen PFS2 events occurred, including 6 in the direct-ASCT2 group and 7 in the re-induction group. Median PFS2 was 25.8 months (95% CI, 1.2–50.3) in the direct-ASCT2 group and 11.4 months (95% CI, 8.3–14.5) in the re-induction group (log-rank *p* = 0.604). In the univariable Cox model, re-induction was not significantly associated with PFS2 compared with direct ASCT2 (HR 1.36, 95% CI 0.42–4.47; *p* = 0.608).

Ten deaths occurred in this subset, including 4 in the direct-ASCT2 group and 6 in the re-induction group. Median OS was 48.2 months (95% CI, 39.3–57.1) and 39.6 months (95% CI, 10.5–68.6), respectively. The survival distributions differed in the log-rank analysis (*p* = 0.048); however, the corresponding univariable Cox estimate was highly imprecise and did not reach conventional statistical significance (HR 4.97, 95% CI 0.91–27.16; *p* = 0.065). Given the small subgroup size, sparse high-risk abnormalities, and wide confidence intervals, these findings were considered exploratory.

### 3.6. Summary of Multivariable Analyses

Across both models, no clinical variable, including pre-ASCT2 strategy, ISS stage, or early relapse, showed a statistically significant independent association with PFS2 or OS. However, these findings should be interpreted cautiously given the limited sample size and number of events (Table 3 and Table 4).

## 4. Discussion

In this multicenter real-world cohort of patients with relapsed MM undergoing non-tandem ASCT2, pre-transplant re-induction chemotherapy was not associated with a significant improvement in PFS2 compared with proceeding directly to ASCT2. Although unadjusted OS appeared longer in the direct-ASCT2 group, this association was no longer statistically significant after adjustment for ISS stage and early relapse after ASCT1. These findings suggest that the apparent survival differences between pre-ASCT2 strategies are likely influenced by treatment selection and clinical context rather than by the sequencing strategy alone.

ASCT2 remains an important salvage strategy for selected patients with relapsed multiple myeloma, particularly for those who previously derived meaningful benefit from first autologous transplantation. In line with prior institutional series and registry-based analyses, our multicenter cohort demonstrates that ASCT2 can provide clinically relevant disease control, with a median PFS2 of approximately 20 months and an overall survival approaching four years, findings that are broadly consistent with contemporary ASCT2 cohorts [3,4,5,6,7,8,9]. These findings reinforce the continued role of ASCT2 as a feasible and effective therapeutic option in routine clinical practice.

A central and unresolved question in the management of relapsed myeloma concerns the optimal sequencing of therapy before ASCT2. In real-world settings, some patients proceed directly to transplantation at relapse, whereas others receive re-induction chemotherapy with the intent of cytoreduction or response deepening prior to high-dose melphalan. However, comparative evidence addressing whether this additional pre-transplant therapy translates into improved post-ASCT2 outcomes remains limited. The study by Miller et al. represents the most directly relevant comparative evidence regarding re-induction before salvage autologous transplantation. In that retrospective cohort, re-induction was not associated with improved post-transplant time to next therapy or OS compared with proceeding directly to transplantation. Although their population was not restricted exclusively to patients undergoing ASCT2 after a previous transplant, their findings support the possibility that routine re-induction may not provide an independent survival advantage. Our results extend this observation to a multicenter cohort composed specifically of patients undergoing a true second autologous transplantation. Nevertheless, both studies remain vulnerable to confounding by indication and cannot establish the equivalence of the two strategies [10]. Previous retrospective series, comparative studies, and the randomized Myeloma X trial have supported the clinical value of salvage autologous transplantation in appropriately selected patients [11,12,13,14,15]. However, these studies did not specifically compare direct ASCT2 with re-induction followed by ASCT2.

Importantly, the relatively small sample size of the present cohort should be interpreted in the context of ASCT2 practice in relapsed MM. Unlike frontline ASCT, ASCT2 is applied only to a selected subset of patients who remain transplant-eligible at relapse, have adequate organ function and performance status, and are considered likely to benefit from high-dose melphalan re-challenge. Consequently, many real-world ASCT2 series, particularly those addressing specific clinical questions such as treatment sequencing before ASCT2, include modest patient numbers [3,5,6,8,9]. In this regard, our cohort size is consistent with the limited but clinically relevant nature of the available ASCT2 literature, and the multicenter design helps mitigate the limitations of single-institution experience.

Within this context, our study provides real-world insight into contemporary practice patterns across multiple centers. In our cohort, re-induction chemotherapy prior to ASCT2 was not associated with a measurable improvement in progression-free survival after transplantation. Although unadjusted analyses demonstrated numerically shorter overall survival among patients receiving re-induction, this difference did not persist after multivariable adjustment, suggesting that baseline clinical factors and treatment allocation, rather than the pre-ASCT2 strategy itself, likely influenced observed outcomes.

Nevertheless, treatment allocation was not randomized, and confounding by indication remains an important consideration. Patients selected for re-induction may have had symptomatic relapse, higher disease burden, more rapid relapse kinetics, a perceived need for immediate cytoreduction, or other adverse clinical or biological characteristics that were not systematically captured in the retrospective dataset. Because the specific reasons for treatment allocation were not uniformly documented at the patient level, adjustment for ISS stage and PFS1 could not fully account for these differences. Although all patients in the direct-ASCT2 group had progressive disease immediately before transplantation, the PD category does not quantify the magnitude or clinical consequences of progression. The distinction between biochemical and clinical relapse, symptom burden, tumor burden, and relapse kinetics was not uniformly documented. Other incompletely recorded factors, including cytogenetic risk, MRD status, and subsequent treatment exposures, may also have influenced PFS2 and OS. Accordingly, the observed associations between pre-ASCT2 strategy and survival outcomes should not be interpreted as causal effects of either treatment approach.

Another notable observation from our analysis is the similarity in post-ASCT2 response rates between the two groups, despite differences in pre-transplant management. This finding underscores the dominant cytotoxic effect of high-dose melphalan conditioning in the salvage setting and suggests that, once patients proceed to ASCT2, the depth of response achieved before transplantation may exert a limited incremental influence on early post-transplant outcomes. The low transplant-related mortality observed in our cohort further supports the feasibility and safety of ASCT2 across diverse clinical scenarios. Maintenance therapy after ASCT2 was administered at similar frequencies in the two treatment groups. Nevertheless, maintenance regimens were heterogeneous, and their potential influence on PFS2 and OS cannot be fully excluded. Because maintenance was a post-transplant exposure and detailed information regarding its initiation, duration, and subsequent modifications was not uniformly incorporated into the analysis, it was not modeled as a fixed baseline covariate. Retrospective studies have suggested that maintenance after ASCT2, particularly lenalidomide-based maintenance, may be associated with improved outcomes; however, these observations remain vulnerable to selection bias and time-dependent treatment effects [9,16]. Although the overall distribution of maintenance classes did not differ significantly between the groups, the small regimen-specific subgroups and the lack of uniformly recorded information regarding treatment initiation, duration, and modification precluded reliable time-dependent or regimen-specific survival analyses. Consequently, residual confounding related to post-ASCT2 maintenance cannot be excluded.

These results should also be interpreted in the context of the evolving therapeutic landscape of relapsed MM. The increasing availability of highly active triplet and quadruplet regimens incorporating proteasome inhibitors, immunomodulatory drugs, and anti-CD38 monoclonal antibodies has transformed treatment algorithms in high-income settings. Nevertheless, access to these therapies remains uneven worldwide, and in many health-care systems, their high cost substantially limits widespread use. Under such real-world constraints, ASCT2 continues to represent an accessible, durable, and cost-effective salvage option for eligible patients, underscoring the ongoing relevance of studies evaluating its optimal integration into treatment pathways.

Several limitations of this study warrant emphasis. The retrospective design, non-randomized treatment allocation, and modest sample size limit causal inference. Because the cohort was restricted to patients who ultimately underwent ASCT2, the analysis was conditional on surviving and remaining transplant-eligible until transplantation. Patients who received salvage systemic therapy but did not proceed to ASCT2 were not systematically captured; therefore, the study cannot estimate the comparative effectiveness of the alternative treatment strategies from the time of relapse. This issue is more appropriately regarded as pre-transplant survivor selection rather than immortal time bias within the post-ASCT2 analyses, because no pre-ASCT2 person-time was included in the PFS2 or OS calculations. An additional post-ASCT2 landmark analysis would not correct selection occurring before transplantation; addressing this limitation would require a relapse-based inception cohort that also included patients who failed to reach ASCT2.

However, the cohort size should be interpreted in light of the clinical context: ASCT2 is not routinely performed in all patients with relapsed MM and is generally restricted to a selected subgroup who remain eligible for salvage transplantation. Therefore, comparable real-world ASCT2 studies frequently report limited sample sizes, particularly when focused on specific treatment-sequencing questions. The limited availability of cytogenetic data represents a major source of residual confounding rather than a minor missing-data issue. FISH results were available for only 18 of 42 patients, and the proportion of patients with available testing differed between the direct-ASCT2 and re-induction groups. Moreover, classical high-risk FISH features were identified in two evaluable patients in the direct-ASCT2 group but in none of the evaluable patients in the re-induction group, whereas 1q gain/amplification was identified in one patient in the re-induction group. A sensitivity analysis restricted to patients with available FISH data did not demonstrate a significant association between pre-ASCT2 treatment strategy and PFS2. Although the log-rank comparison suggested a difference in OS, the corresponding Cox estimate was highly imprecise, with a very wide confidence interval that included the null. The small evaluable subset, limited number of events, and sparse distribution of high-risk abnormalities precluded reliable multivariable adjustment for cytogenetic risk. Therefore, this restricted analysis cannot eliminate the possibility that an unequal distribution of adverse disease biology materially influenced the estimated associations between treatment strategy and survival outcomes. Minimal residual disease assessment was also not uniformly available. In addition, the limited number of PFS2 and OS events resulted in substantial statistical imprecision. The event-based sensitivity analysis indicated that the study had 80% power to detect only very large treatment effects, corresponding to hazard ratios of approximately 3.00 for PFS2 and 5.42 for OS, or their reciprocal effects in the opposite direction. Therefore, the absence of a statistically significant difference in PFS2 should not be interpreted as evidence of equivalence, non-inferiority, or the absence of a clinically meaningful difference between direct ASCT2 and re-induction followed by ASCT2. The wide confidence intervals around the observed treatment-effect estimates remain compatible with potentially meaningful benefit or harm. Despite these limitations, the multicenter design and direct comparison of two clinically relevant pre-ASCT2 strategies provide useful hypothesis-generating data for routine practice.

In summary, our findings suggest that routine re-induction chemotherapy before ASCT2 was not associated with a clear survival advantage in this real-world cohort. However, given the potential for selection bias and unmeasured confounding, these results should be regarded as hypothesis-generating rather than practice-changing. Prospective multicenter studies, well-curated registry analyses, or randomized clinical trials with predefined treatment-selection criteria and standardized follow-up are needed to validate these findings.

## 5. Conclusions

In conclusion, this multicenter real-world analysis did not demonstrate a clear survival advantage for routine re-induction chemotherapy before ASCT2. However, given the retrospective nature of treatment allocation and the potential for selection bias, these results should not be interpreted as evidence against re-induction in individual patients. Rather, they underscore the uncertainty surrounding optimal timing and sequencing before ASCT2 and support the need for prospective or registry-based studies with predefined treatment algorithms.

## Figures and Tables

**Table 1:** Baseline and Disease Characteristics of Patients Undergoing ASCT2.

Characteristic	All Patients (*n* = 42)	Direct ASCT2 (*n* = 21)	ASCT2 After Re-Induction (*n* = 21)
Age at diagnosis, years, median (range)	55.5 (36–67)	56.0 (43–67)	53.0 (36–65)
Age at ASCT2, years, median (range)	58.4 (41–74)	58.7 (46–73)	57.4 (41–74)
Sex, *n* (%)			
Female	25 (59.5)	13 (61.9)	12 (57.1)
Male	17 (40.5)	8 (38.1)	9 (42.9)
ISS stage, *n* (%)			
I	10 (23.8)	5 (23.8)	5 (23.8)
II	20 (47.6)	11 (52.4)	9 (42.9)
III	12 (28.6)	5 (23.8)	7 (33.3)
Conventional cytogenetics, *n* (%)			
Normal karyotype	15 (35.7)	6 (28.6)	9 (42.9)
Complex karyotype	2 (4.8)	1 (4.8)	1 (4.8)
Not available	25 (59.5)	14 (66.7)	11 (52.4)
FISH findings			
FISH data available, *n* (%)	18 (42.9)	7 (33.3)	11 (52.4)
Any FISH abnormality among evaluable patients, *n*/*N* (%)	4/18 (22.2)	2/7 (28.6)	2/11 (18.2)
Classical high-risk FISH feature *, *n*/*N* (%)	2/18 (11.1)	2/7 (28.6)	0/11 (0.0)
1q gain/amplification, *n*/*N* (%)	1/18 (5.6)	0/7 (0.0)	1/11 (9.1)
Isotype, *n* (%)			
IgG	26 (61.9)	15 (71.4)	11 (52.4)
IgA	10 (23.8)	6 (28.6)	4 (19.1)
Light chain only	6 (14.3)	0 (0)	6 (28.6)
Light chain type, *n* (%)			
Kappa	23 (54.8)	11 (52.4)	12 (57.1)
Lambda	19 (45.2)	10 (47.6)	9 (42.9)
Induction before ASCT1, *n* (%)			
VCD	27 (64.3)	15 (71.4)	12 (57.1)
VRD	15 (35.7)	6 (28.6)	9 (42.9)
Best response before ASCT1, *n* (%)			
CR	6 (14.3)	3 (14.3)	3 (14.3)
VGPR	9 (21.4)	5 (23.8)	4 (19.1)
PR	22 (52.4)	10 (47.6)	12 (57.1)
SD	5 (11.9)	3 (14.3)	2 (9.5)
PD	0 (0)	0 (0)	0 (0)
Best response after ASCT1, *n* (%)			
CR	6 (14.3)	3 (14.3)	3 (14.3)
VGPR	21 (50.0)	10 (47.6)	11 (52.4)
PR	11 (26.2)	5 (23.8)	6 (28.6)
SD	0 (0)	0 (0)	0 (0)
PD	4 (9.5)	3 (14.3)	1 (4.8)
Maintenance after ASCT1, *n* (%)			
Yes	13 (31.0)	6 (28.6)	7 (33.3)
No	29 (69.0)	15 (71.4)	14 (66.7)
Type of maintenance therapy after ASCT1 (*n*, %)			
Bortezomib	2 (15.4)	1 (16.7)	1 (14.3)
Lenalidomide	11 (84.6)	5 (83.3)	6 (85.7)
PFS1, months, median (range)	15.4 (2.2–126.2)	10.7 (2.2–126.2)	19.1 (7.9–114.7)
Early relapse after ASCT1 (PFS1 < 18 months), *n* (%)	22 (52.4)	15 (71.4)	7 (33.3)

ASCT1: First autologous stem cell transplantation, ASCT2: Second autologous stem cell transplantation, PFS1: Progression-free survival after ASCT1. * Classical high-risk FISH features were defined as del(17p), t(4;14), or t(14;16). Percentages for maintenance regimen types were calculated among patients who received maintenance after ASCT1. Early relapse after ASCT1 was defined as PFS1 <18 months. The between-group distribution of best response before ASCT1 was compared using the Fisher–Freeman–Halton exact test (*p* = 0.934); the PD category was not included in the test because no patients were classified as PD in either group.

**Table 2:** Clinical Characteristics Before ASCT2 and Post-Transplant Outcomes.

Characteristic	All (*n* = 42)	Direct-ASCT2 (*n* = 21)	ASCT2 After Re-Induction (*n* = 21)
Number of re-induction lines before ASCT2, *n* (%)			
1	—	—	16 (76.2)
2	—	—	2 (9.5)
3	—	—	3 (14.3)
Disease status immediately before ASCT2, *n* (%)			
CR	1 (2.4)	0 (0.0)	1 (4.8)
VGPR	9 (21.4)	0 (0.0)	9 (42.9)
PR	4 (9.5)	0 (0.0)	4 (19.0)
SD	0 (0.0)	0 (0.0)	0 (0.0)
PD	28 (66.7)	21 (100.0)	7 (33.3)
Interval between relapse and ASCT2, months, median (range)	22.9 (3.0–132.3)	5.2 (3.0–17.6)	35.8 (14.0–132.3)
Best response after ASCT2, *n* (%)			
CR	5 (11.9)	2 (9.5)	3 (14.3)
VGPR	21 (50.0)	10 (47.6)	11 (52.4)
PR	13 (31.0)	7 (33.3)	6 (28.6)
PD	3 (7.1)	2 (9.5)	1 (4.8)
Maintenance after ASCT2, *n* (%)			
Yes	25 (59.5)	13 (61.9)	12 (57.1)
No	17 (40.5)	8 (38.1)	9 (42.9)
Type of maintenance therapy after ASCT2, *n* (%)			
Proteasome inhibitors	9 (36.0)	6 (46.2)	3 (25.0)
Immunomodulators	12 (48.0)	5 (38.5)	7 (58.3)
Anti-CD38 antibody	4 (16.0)	2 (15.4)	2 (16.7)
PFS2 event (progression or death), *n* (%)	26 (61.9)	16 (76.2)	10 (47.6)
Status at last follow-up, *n* (%)			
Alive	31 (73.8)	17 (80.9)	14 (66.7)
Deceased	11 (26.2)	4 (19.1)	7 (33.3)
TRM within 100 days, *n* (%)	1 (2.4)	0	1 (4.8)

ASCT1: First autologous stem cell transplantation, ASCT2: Second autologous stem cell transplantation, TRM: Transplantation-related mortality, —: not applicable. The number of re-induction lines was applicable only to patients who received re-induction therapy before ASCT2. Disease status immediately before ASCT2 was based on the last available IMWG assessment before conditioning. All patients in the direct-ASCT2 group proceeded to transplantation with progressive disease without intervening systemic therapy. Percentages for maintenance regimen types were calculated among patients who received maintenance therapy after ASCT2.

**Table 3:** Multivariable Cox Regression Analysis for PFS2.

Variable	HR	95% CI	*p*-Value
Re-Induction vs. Direct ASCT2	1.17	0.45–3.07	0.751
PFS1 < 18 months	1.10	0.39–3.06	0.858
ISS Stage	-	-	0.632
ISS II vs. I	0.62	0.19–2.03	0.429
ISS III vs. I	0.66	0.26–1.68	0.381

**HR:** Hazard Ratio, **CI:** Confidence Interval.

**Table 4:** Multivariable Cox Regression Analysis for OS.

Variable	HR	95% CI	*p*-Value
Direct ASCT2 vs. Re-Induction	0.41	0.11–1.45	0.164
PFS1 < 18 months	0.74	0.24–2.30	0.604
ISS Stage	-	-	0.216
ISS II vs. I	0.37	0.10–1.42	0.149
ISS III vs. I	0.45	0.17–1.22	0.115

**HR:** Hazard Ratio, **CI:** Confidence Interval.

## Data Availability

De-identified data may be made available from the corresponding author upon reasonable request and with appropriate ethical approval. Public sharing of the dataset is restricted due to patient confidentiality regulations.

## Abbreviations

ASCT, autologous stem cell transplantation; ASCT1, first autologous stem cell transplantation; ASCT2, second autologous stem cell transplantation; CI, confidence interval; CIBMTR, Center for International Blood and Marrow Transplant Research; CR, complete response; FISH, fluorescence in situ hybridization; HR, hazard ratio; IMiD, immunomodulatory drug; IMWG, International Myeloma Working Group; ISS, International Staging System; MM, multiple myeloma; MRD, minimal residual disease; ORR, overall response rate; OS, overall survival; PD, progressive disease; PFS, progression-free survival; PFS1, progression-free survival after ASCT1; PFS2, progression-free survival after ASCT2; PR, partial response; SD, stable disease; TRM, transplant-related mortality; VCD, bortezomib, cyclophosphamide, and dexamethasone; VGPR, very good partial response; VRD, bortezomib, lenalidomide, and dexamethasone.
